# Identifying drinking patterns in a middle-aged population and their association with coronary heart disease, stroke, and all-cause mortality risk: the Danish Health Examination Survey

**DOI:** 10.1093/eurpub/ckag162

**Published:** 2026-09-02

**Authors:** Marta Trius-Soler, Marta Guasch-Ferré, Cristina Valle-Hita, Majken K Jensen, Janne S Tolstrup

**Affiliations:** Section of Epidemiology, Department of Public Health, Faculty of Health and Medical Sciences, University of Copenhagen, Copenhagen, Denmark; Novo Nordisk Foundation Center for Basic Metabolic Research, Faculty of Health and Medical Sciences, University of Copenhagen, Copenhagen, Denmark; National Institute of Public Health, University of Southern Denmark, Copenhagen, Denmark; Section of Epidemiology, Department of Public Health, Faculty of Health and Medical Sciences, University of Copenhagen, Copenhagen, Denmark; Novo Nordisk Foundation Center for Basic Metabolic Research, Faculty of Health and Medical Sciences, University of Copenhagen, Copenhagen, Denmark; Department of Nutrition, Harvard TH Chan School of Public Health, Boston, MA, United States; Section of Epidemiology, Department of Public Health, Faculty of Health and Medical Sciences, University of Copenhagen, Copenhagen, Denmark; Novo Nordisk Foundation Center for Basic Metabolic Research, Faculty of Health and Medical Sciences, University of Copenhagen, Copenhagen, Denmark; Section of Epidemiology, Department of Public Health, Faculty of Health and Medical Sciences, University of Copenhagen, Copenhagen, Denmark; Department of Nutrition, Harvard TH Chan School of Public Health, Boston, MA, United States; National Institute of Public Health, University of Southern Denmark, Copenhagen, Denmark

## Abstract

Alcohol drinking patterns encompass various dimensions of consumption. However, most studies have focused narrowly on the quantity of alcohol consumed. This study aimed to identify alcohol-drinking patterns in middle-aged population using alcohol-related variables and to examine their relationship with coronary heart disease (CHD), stroke, and all-cause-mortality risk. The present population-based study included adults from the Danish Health Examination Survey (DANHES) cohort who were ≥40 years old and free of CHD and stroke at baseline (*n* = 46 141). Latent Class Analysis (LCA) was used to identify distinct subpopulations based on six alcohol-related variables (amount, frequency, binge episodes, type, within meals, at home and alone). Cox proportional hazard models were used to estimate hazard ratios (95% confidence interval) between drinking patterns and CHD, stroke, and all-cause mortality during a median follow-up of 11.8 years. Four classes of drinking patterns were identified in both sexes (labeled as “low drinkers,” “low-to-moderate drinkers,” “moderate-regular drinkers,” and “frequent-heavy drinkers”). The more frequent drinking pattern was a median of 3 (2–5) drinks per week. Frequent heavy drinking was associated with a higher risk of stroke. Individuals in the low-to-moderate male drinking class and the moderate-regular female drinking class not under heart medication had a lower risk of all-cause mortality than low-level drinkers. Four distinct drinking patterns were identified. Among individuals 40 years and older, frequent-heavy drinking was associated with a higher risk of stroke, while low-to-moderate regular drinking might be associated with a lower all-cause mortality risk compared to low drinkers.

## Introduction

The term drinking pattern refers to the overall manner in which alcohol is consumed, encompassing several dimensions such as the amount and frequency of alcohol intake, the type of beverage, the context of consumption, and the presence of binge drinking episodes. Binge drinking, commonly defined as consuming 5 or more beverages per occasion, and heavy alcohol intake (>60 g/day for men and >40 g/day for women) are well-established risk factors for death and disability, including cardiovascular diseases (CVD) [1, 2]. In contrast, low-to-moderate regular drinking has been reported to exhibit a J-shaped relationship with coronary heart disease (CHD) [3] and all-cause mortality in the long-term among middle-aged or older individuals [1]. However, this J-shaped relationship has been widely debated, with concerns related to potential confounding from the choice of reference group, population age, reverse causation, residual confounding, and the lack of long-term, large-scale randomized controlled trials [4–6].

In this context, the 2021 European Society of Cardiology (ESC) Guidelines on Cardiovascular Disease Prevention in Clinical Practice advocate for the adoption of a healthy diet, restricting alcohol consumption to a maximum of 100 g per week for both male and female drinkers [7]. At the national level, the Danish Health Authority’s 2022 “10-4” recommends a maximum of 10 drinks (120 g of alcohol) per week and no more than 4 drinks per occasion, corresponding to an estimated 1%–2% risk of alcohol-related death [8]. A 2025 National Academies report found that, compared with lifetime abstainers, moderate alcohol consumption is associated with lower risk of nonfatal myocardial infarction and stroke (low certainty), and CVD mortality (moderate certainty) [9].

Previous studies have investigated drinking patterns dimensions through stratified analyses, revealing differences in health effects based on specific alcoholic beverages [10–12], mean alcohol consumption trajectory profiles [13], the combination of some of the drinking pattern dimensions [14–17] or even the adherence to a Mediterranean drinking pattern relying on predefined categories and composite score [18]. Despite these contributions, most studies have focused narrowly on alcohol quantity (i.e. g/day) [19]. These conventional approaches rely on researcher-defined assumptions based on prior evidence, which may not reflect real-world drinking patterns. In contrast, LCA is a data-driven, person-centered method that identifies distinct subgroups based on observed behavior patterns [20]. In addition, irrespective of the study design, sex differences in important risk factors for CVD have also been described [21] as well as age-related and sex-specific differences in drinking behavior [22].

To address these gaps, we have used a comprehensive approach that includes several dimensions of individuals’ drinking patterns, applied specific definitions for both lifetime and current abstainers, accounted for several potential confounders, described some dietary pattern components, and stratified by sex within a middle-aged Danish cohort from the general population. Specifically, this study aims to identify distinct alcohol drinking patterns using a set of alcohol-related variables by applying an LCA. Additionally, it seeks to examine the relationship between drinking patterns and CHD, stroke, and all-cause mortality risk.

## Methods

### Survey design and cohort description

The Danish Health Examination Survey (DANHES) from 2007 to 2008 was a survey conducted in 13 of 98 municipalities in Denmark, based on geographical representation [23]. A total of 538 497 adults aged ≥18 years were invited by letter to complete two self-reported questionnaires. From those invited, 76 484 individuals participated (14%). The study protocol was approved by the Ethics Committee for the Capital Region of Denmark (journal nos: H-A-2008-126 and H-C-2007-0118) and conducted with compliance with the Declaration of Helsinki. All participants signed an informed consent.

### Alcohol drinking dimensions assessment

Twelve questions related to alcohol consumption were included in DANHES-questionnaire, and used to define six drinking dimensions:

#### Drinking status

Non-drinkers were categorized as lifetime abstainers, indicating individuals who had never consumed alcohol. Current abstainers referred to individuals with a history of alcohol consumption but had refrained from drinking within the last year. Drinkers were individuals who had consumed alcohol in the past year and were further queried about their drinking patterns.

#### Amount, frequency, and type of alcohol

Beverage-specific consumption (beer, wine, sweet wine, and spirits) for each day in a typical week was recorded in drinks (12 g ethanol/drink). The overall average weekly alcohol amount was calculated based on the average weekly consumption of the reported specific alcoholic beverages. Additionally, information on the frequency of days consuming alcohol was reported as: <1; 1–2; 3–4; or 5–7 days/week.

#### Within meals, at home and alone, and binge episodes

To contextualize alcohol consumption, participants reported the proportion of their alcohol consumption that occurred with meals and the typical situation of alcohol consumption. Alcohol consumption within meals rate was coded as: never; a quarter; half; three-quarters; or always. Drinking situation was classified as “at home and alone” (yes, no) based on the participant’s selection from a battery of multi-answer options. Participants were also queried about binge drinking frequency (defined as consuming ≥5 beverages on one occasion), with responses categorized as: never; <1 day/month; 1–3 days/month, 1 day/week, or >1 day/week.

Participants were also asked to indicate whether their alcohol consumption had decreased, increased, or remained stable over the last 5 years.

### Incidence of CHD and stroke, deaths, and migrations assessment

Information from participants was obtained from the Danish National Health Service Registries from 1st of January of 1997 to 31st of December 2019. Information on CHD and stroke was obtained from the Danish Patient Registry, which comprises all incident cases diagnosed in Denmark. Cases were assigned according to the World Health Organization’s International Classification of Diseases (ICD) 10th revision diagnosis criteria for any CHD (ICD 10 codes I20-I25) and stroke (ICD 10 codes I60-I64 and G459). Hemorrhagic stroke was assigned if ICD-10 code was I60-I61. Deaths and migrations during follow-up were obtained from the Danish Civil Registration System.

### Covariates assessment

Information on sociodemographic, lifestyle, health status, and medication was recorded through a self-reported DANHES questionnaire, except for age and sex that were obtained from the Danish Civil Registration System. Danish ethnicity was defined as both parents born in Denmark. Level of school education was defined according to the International Standard Classification of Education, combining ongoing or completed school and vocational education. Occupational status and married or living with a partner (yes/no) were also recorded. Smoking was reported as status, amount of daily smoking (in number of daily cigarettes, cheroots, cigars, and pipes), daily smoking duration (in years), and time since quitting daily smoking (in years). Information on diet and physical activity methods are described in a previous report [14, 24]. Participants were classified as having (yes/no or missing) chronic illness, diabetes, hypertension, or taking blood pressure-lowering and heart medication based on self-reporting data. Body mass index (BMI) was calculated through self-reported height (cm) and weight (kg). Among females, we used information on menstruation to define their menopausal status (peri- and post-menopausal/pre-menopausal women/not known or missing) and hormone replacement therapy medication (yes, now/yes, earlier/never or missing).

### Selection of the final study population

Participants with no information about their alcohol consumption in the past year, missing data on all drinking pattern dimensions, and on both the amount and frequency of alcohol consumption were excluded (Supplementary Fig. S1). Prior to exclusions based on missing alcohol consumption data, conflicting responses between the total amount of alcohol consumption, drinking frequency, and binge drinking episodes were reviewed. A threshold of 30 or more drinks per day was considered unreliable, using the ratio between the total amount per week of alcohol intake and the higher end of the frequency category reported. Alcohol consumption amounts were treated as missing for participants whose responses met this criterion (*n* = 119).

Analysis was restricted to middle-aged individuals (≥40 years old) due to the no safe level of alcohol consumption for younger people, the strong age dependency of drinking patterns, and the need for additional evidence on the ongoing debate about the potential cardiovascular benefits of moderate alcohol consumption among middle-aged adults [6]. Pregnant females at baseline or those who had given birth within the last 6 months from baseline were excluded. We also excluded participants with a diagnosis of CHD or stroke at baseline. Subjects with incomplete data on any potential confounder included in the Cox-proportional hazard regression model 2, as well as BMI, were excluded to maintain a harmonized analytic dataset and facilitate interpretability (8.9% excluded due to missingness). The final numbers of missing data were 94 (amount), 123 (frequency), 61 (binge drinking), 70 (type), 123 (within meals), and 87 (at home and alone).

### Statistical analysis

We employed LCA to identify distinct subpopulations considering six drinking dimensions, restricting the analysis to individuals aged 40 years or older and stratifying by sex. The remaining variables were used only to define comparison groups. For LCA models, drinking dimensions were simplified by reducing the number of categories and considering only drinkers: (i) *Amount* as <7, 7–13.9, 14–20.9, ≥21 drinks/week; (ii) *Frequency* as <1, 1–2, 3–4, 5–7 days/week; (iii) *Type* as beer or wine drinkers if ≥50% of their total alcohol consumption came from that specific beverage type. Sweet wine was considered as wine. Participants not fitting into either category were classified as mixed drinkers; (iv) *Within meals* as a binary (yes/no) variable indicating whether 100% of alcohol consumption occurred during meals; (v) *At home and alone* a binary (yes/no) variable based on participants’ selection from a multi-option response set; (vi) *Binge drinking* was defined as ≥1 episode/month (yes/no). Lifetime abstainers and current abstainers were not added in the LCA models.

Several models were tested with varying numbers of latent classes (e.g. 2-class, up to 6-class models) to determine the best fit for both males and females. The optimal number of classes was identified using the Bayesian Information Criterion (BIC) and Akaike Information Criterion (AIC). A lower BIC or AIC indicates a better model fit, while entropy values closer to 1 suggests more distinct classification. Class structures were compared based on response patterns, class count and size, with final selection guided by theoretical interpretability and practical significance. Four latent classes were identified as the optimal categories for both sexes (Supplementary Table S1 and Figs S2 and S3). After determination of the final latent class model, each individual was assigned to the class to which they had the highest posterior probability of belonging given their alcohol drinking dimensions.

Baseline characteristics were described with median values and interquartile range (IQR) for continuous variables and number and proportions for categorical variables. The relationship between drinking patterns classes and CHD, stroke, and all-cause mortality risk were studied using Cox-proportional hazard regression models. The low drinkers were used as the reference group. Age (in days) was used as the underlying time axis, and analyses were corrected considering birth date and study entry date. Time was measured as the duration from the participant’s entry into the study until the occurrence of the event or censoring (death, migration, or end of follow-up) (Supplementary Fig. S1). Analyses were carried out by sex and for the entire cohort and adjusted by several confounders. Model 3 was adjusted for confounders that could also be potential mediators. Sex-drinking pattern interaction analysis was assessed using log likelihood test comparing models with and without the interaction term. Sensitivity analyses were conducted, including only participants who reported stable alcohol consumption over the previous 5 years (74.4%), those not taking heart medication (*n* = 43 781), or excluding data from the first 2 years after the questionnaire was administered. Residual confounding coming from smoking was studied by running the analysis on the never-smokers, and results were compared with Model 3 (fully adjusted, excluding smoking).

All statistical analyses were performed in STATA statistical software version 18.0 (Stata Corp, College Station, TX, USA) and SAS software version 9.4 (SAS Institute, Cary, NC, USA). RStudio was used to build figures (http://www.rstudio.com). Statistical tests were two-sided and *P*-values below .05 were considered significant.

## Results

The final study sample included 46 141 individuals. A total of 2752 CHD events, 2086 stroke events, and 3616 deaths occurred during a median (IQR) follow-up of 11.8 (11.3, 12.2) years. 274 individuals experimented both events. Among stroke events, 229 (11.0%) were hemorrhagic strokes. In total, 1% participants were lifetime abstainers (*n* = 443), 3% current abstainers (*n* = 1381), and 96% drinkers (*n* = 44 317).

### Drinking pattern latent classes

**Figure 1:**
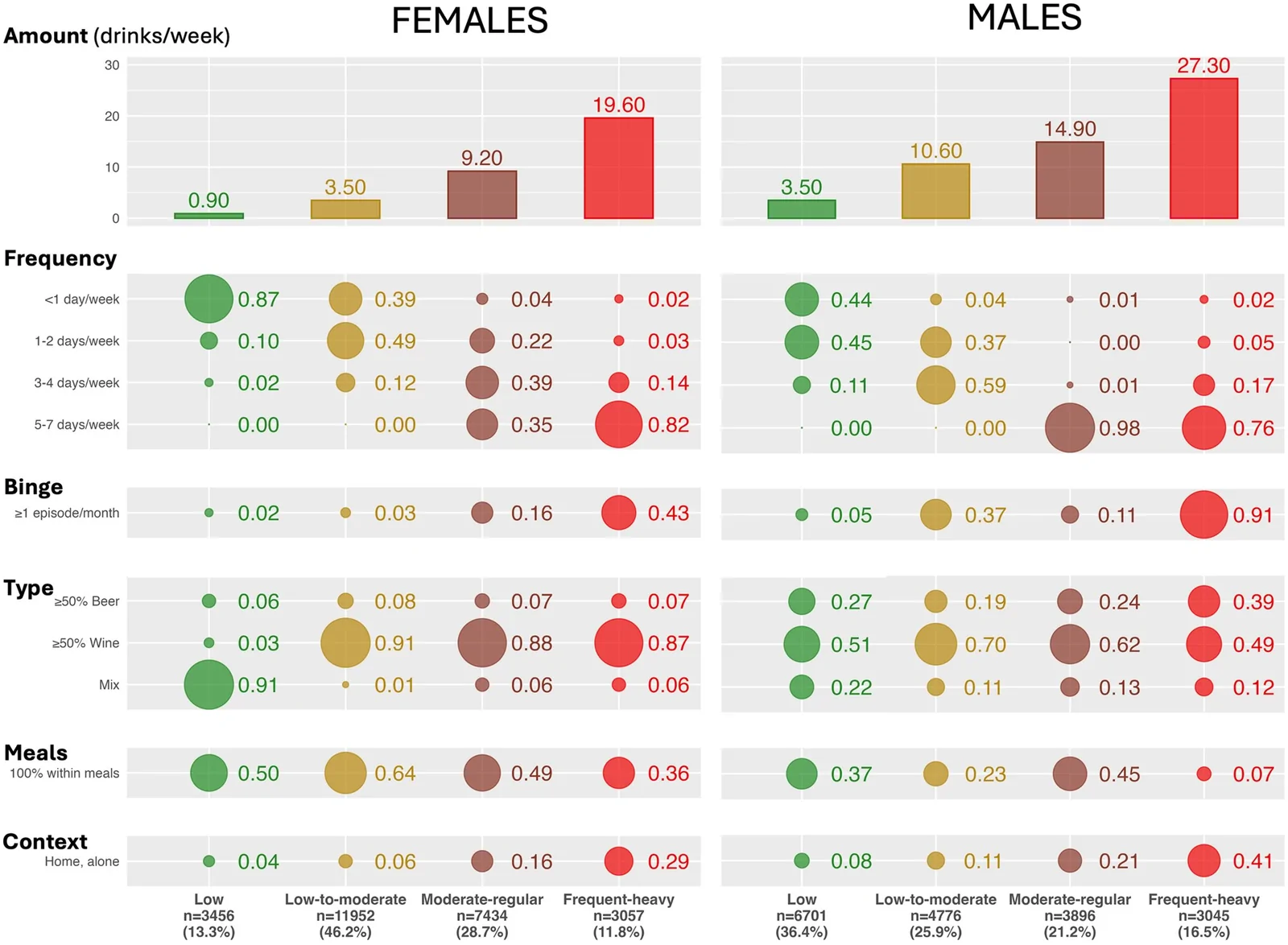
Drinking pattern characteristics across latent classes by sex. Each point on the graph represents the likelihood (probability, ranging from 0 to 1) for each dimension within each Class. The bars represent to the absolute frequency (count). One standard drink corresponding to 12 g ethanol. *Note*: Lifetime and current abstainers were excluded from this analysis but treated as two separate additional classes.

A 4-class model provided the most comprehensive and parsimonious model. Low drinkers (13.3% females, 35.4% males) consumed small amounts infrequently, rarely drank alone or at home, and showed no preference for beverage type. Low-to-moderate drinkers (46.2% females, 25.9% males) drank more frequently, primarily wine, but had low binge-drinking rates. Moderate-regular drinkers (28.7% females, 21.2% males) consumed higher amounts of alcohol and more frequently (98% of males drank 5–7 days/week) but followed similar patterns to low-to-moderate drinkers. Frequent-heavy drinkers (11.8% females, 16.5% males) had the highest weekly consumption (high amount and frequency) and often drank alone without meals. Although binge drinking occurred at low levels in other classes, this class had by far the highest probability (43% females; 91% males), indicating that binge drinking is a defining characteristic of this group. Females in this class preferred wine, while males were split between beer and wine (Fig. 1 and Supplementary Table S2).

### Study population characteristics by drinking latent classes

Table 1 shows the baseline characteristics by sex and drinking latent classes. The median (IQR) age was 56 years (48–63), with 58.9% females. Females consumed vegetables, fruits, and engaged in physical activity more frequently than males. Among females, 55.9% were peri- or postmenopausal, 26.0% premenopausal, and 22.1% were uncertain or had missing data. Median (IQR) alcohol consumption was 5 (2–9) drinks/week [60 (24–108) g of alcohol/week] for females and 9 (4–15) drinks/week [108 (48–180) g of alcohol/week] for males. Low-to-moderate and moderate-regular drinkers had healthier diet and were less likely to smoke. Frequent-heavy drinking and current abstainer males were more likely to report hypertension and be current smokers. Those with moderate to high alcohol consumption were more often married or living with a partner, while male moderate-regular drinkers and female abstainers were less likely to be employed. Furthermore, lifetime abstainers were less likely to have attained a high level of education, have both parents born in Denmark, or be current or former smokers. The current abstainers included a higher proportion of individuals who were retired, current smokers, had diabetes, or other chronic illnesses.

**Table 1:** Baseline characteristics of the DANHES study population across drinking pattern latent classes (*n* = 46 141).

	Total	Non-drinkers		Drinkers			
		Lifetime abstainers	Current abstainers	Low	Low-to-moderate	Moderate- regular	Frequent-heavy
Females, *n*	27 170	344	927	3456	11 952	7434	3057
Age, years	55 (48–63)	60 (49–70)	56 (48–64)	54 (46–63)	53 (46–61)	56 (49–63)	59 (52–65)
Sociodemographic, *n* (%)							
Married or living with a partner	21 068 (77.5)	236 (68.6)	604 (65.2)	2357 (68.2)	9452 (79.1)	6051 (81.4)	2368 (77.5)
Danish ethnicity	25 251 (94.1)	245 (74.2)	831 (90.2)	3213 (94.4)	11 158 (94.5)	6973 (94.7)	2831 (94.1)
Education > 14 years	11 452 (42.1)	90 (26.2)	305 (32.9)	1119 (32.4)	4937 (41.3)	3527 (47.4)	1474 (48.2)
Occupation							
Employed	17 664 (65.0)	143 (41.6)	430 (46.4)	2076 (60.1)	8488 (71.0)	4845 (65.2)	1682 (55.0)
Retired	7929 (29.2)	160 (46.5)	401 (43.3)	1105 (32.0)	2809 (23.5)	2245 (30.2)	1209 (39.5)
Other	1577 (5.8)	41 (11.9)	96 (10.4)	275 (8.0)	655 (5.5)	344 (4.6)	166 (5.4)
Lifestyle, *n* (%)							
Smoking							
Current smokers	4387 (16.1)	37 (10.8)	204 (22.0)	736 (21.3)	1634 (13.7)	1098 (14.8)	678 (22.2)
Former smokers	9369 (34.5)	53 (15.4)	311 (33.5)	1061 (30.7)	3699 (30.9)	2863 (38.5)	1382 (45.2)
Never smokers	13 414 (49.4)	254 (73.8)	412 (44.4)	1659 (48.0)	6619 (55.4)	3473 (46.7)	997 (32.6)
^a^Daily smoking duration, years	24 (14–34)	28 (16–38)	30 (18–37)	27 (17–35)	22 (12–31)	22 (12–33)	28 (15–37)
^b^Time since quitting, years	14 (6–24)	13 (4–25)	11 (4–23)	12 (4–22)	14 (6–23)	15 (6–24)	14 (5–24)
Infrequent eaters							
Boiled vegetables	10 327 (38.0)	164 (47.7)	401 (43.3)	1534 (44.4)	4479 (37.5)	2596 (34.9)	1153 (37.7)
Raw vegetables	12 344 (45.4)	198 (57.6)	518 (55.9)	1879 (54.4)	5191 (43.4)	3099 (41.7)	1459 (47.7)
Fruits	3398 (12.5)	63 (18.3)	171 (18.4)	567 (16.4)	1151 (9.6)	830 (11.2)	616 (20.2)
Fiber-rich bread or grain	2772 (10.2)	66 (19.2)	157 (16.9)	478 (13.8)	1077 (9.0)	657 (8.8)	337 (11.0)
Fish	12 444 (45.8)	164 (47.7)	468 (50.5)	1854 (53.6)	5746 (48.1)	3097 (41.7)	1115 (36.5)
MVPA	21 675 (79.8)	295 (85.8)	799 (86.2)	2900 (83.9)	9365 (78.4)	5813 (78.2)	2503 (81.9)
Health status, *n* (%)							
BMI, kg/m^2^	24.0 (21.9–26.8)	25.4 (22.0–28.1)	24.4 (22.0–28.2)	25.0 (22.5–28.7)	24.2 (22.0–27.0)	23.6 (21.6–26.0)	23.8 (21.7–26.0)
Hypertension	6810 (25.1)	117 (34.0)	275 (29.7)	954 (27.6)	2837 (23.7)	1768 (23.8)	859 (28.1)
Diabetes	790 (2.9)	15 (4.4)	74 (8.0)	143 (4.1)	316 (2.6)	163 (2.2)	79 (2.6)
Chronic illness	9519 (35.0)	167 (48.5)	486 (52.4)	1445 (41.8)	3973 (33.2)	2389 (32.1)	1059 (34.6)
Peri- and postmenopausal	14 109 (51.9)	203 (59.0)	507 (54.7)	1646 (47.6)	5673 (47.5)	4115 (55.4)	1965 (64.3)
Current medication, *n* (%)							
Lowering blood pressure	4879 (18.0)	88 (25.6)	208 (22.4)	717 (20.7)	2004 (16.8)	1244 (16.7)	618 (20.2)
Heart	1068 (3.9)	27 (7.8)	52 (5.6)	188 (5.4)	401 (3.4)	257 (3.5)	143 (4.7)
Hormone therapy	1509 (5.6)	14 (4.1)	57 (6.1)	168 (4.9)	582 (4.9)	462 (6.2)	226 (7.4)
Males, *n*	18 971	99	454	6701	4776	3896	3045
Age, years	57 (49–65)	56 (45–66)	57 (50–63)	54 (46–63)	55 (48–63)	63 (56–69)	57 (50–64)
Sociodemographic, *n* (%)							
Married or living with a partner	16 192 (85.4)	79 (79.8)	318 (70.0)	5697 (85.0)	4254 (89.1)	3409 (87.5)	2435 (80.0)
Danish ethnicity	17 609 (93.8)	66 (69.5)	388 (87.6)	6208 (93.5)	4495 (94.7)	3630 (94.3)	2822 (94.0)
Education > 14 years	8040 (42.4)	26 (26.3)	159 (35.0)	2468 (36.8)	2121 (44.4)	1913 (49.1)	1353 (44.4)
Occupation							
Employed	12 531 (66.1)	62 (62.6)	243 (53.5)	4739 (70.7)	3590 (75.2)	1853 (47.6)	2044 (67.1)
Retired	5908 (31.1)	30 (30.3)	174 (38.3)	1756 (26.2)	1087 (22.8)	1960 (50.3)	901 (29.6)
Other	532 (2.8)	7 (7.1)	37 (8.1)	206 (3.1)	99 (2.1)	83 (2.1)	100 (3.3)
Lifestyle, *n* (%)							
Smoking							
Current smokers	3495 (18.4)	14 (14.1)	113 (24.9)	1096 (16.4)	722 (15.1)	688 (17.7)	862 (28.3)
Former smokers	7695 (40.6)	24 (24.2)	186 (41.0)	2329 (34.8)	1931 (40.4)	1864 (47.8)	1361 (44.7)
Never smokers	7781 (41.0)	61 (61.6)	155 (34.1)	3276 (48.9)	2123 (44.5)	1344 (34.5)	822 (27.0)
^a^Daily smoking duration, years	27 (17–38)	23 (17–34)	30 (20–40)	26 (16–37)	24 (15–35)	30 (18–41)	30 (20–39)
^b^Time since quitting, years	15 (7–26)	12 (4–26)	11 (5–22)	14 (6–25)	16 (7–26)	19 (8–30)	14 (6–24)
Infrequent eaters							
Boiled vegetables	10 453 (55.1)	67 (67.7)	258 (56.8)	3805 (56.8)	2600 (54.4)	1978 (50.8)	1745 (57.3)
Raw vegetables	11 845 (62.4)	72 (72.7)	301 (66.3)	4295 (64.1)	2818 (59.0)	2309 (59.3)	2050 (67.3)
Fruits	6064 (32.0)	26 (26.3)	159 (35.0)	1957 (29.2)	1355 (28.4)	1226 (31.5)	1341 (44.0)
Fiber-rich bread or grain	3001 (15.8)	26 (26.3)	96 (21.1)	1071 (16.0)	745 (15.6)	408 (10.5)	655 (21.5)
Fish	9134 (48.1)	47 (47.5)	235 (51.8)	3602 (53.8)	2274 (47.6)	1536 (39.4)	1440 (47.3)
MVPA	12 863 (67.8)	75 (75.8)	346 (76.2)	4554 (68.0)	3025 (63.3)	2761 (70.9)	2102 (69.0)
Health status, *n* (%)							
BMI, kg/m^2^	25.6 (23.7–27.8)	25.2 (23.3–28.7)	25.4 (23.5–28.7)	25.7 (23.7–28.1)	25.7 (23.9–27.8)	25.2 (23.4–27.2)	26.0 (24.1–28.4)
Hypertension	5036 (26.5)	21 (21.2)	139 (30.6)	1570 (23.4)	1155 (24.2)	1142 (29.3)	1009 (33.1)
Diabetes	954 (5.0)	11 (11.1)	43 (9.5)	343 (5.1)	178 (3.7)	219 (5.6)	160 (5.3)
Chronic illness	5896 (31.1)	30 (30.3)	209 (46.0)	2109 (31.5)	1323 (27.7)	1252 (32.1)	973 (32.0)
Current medication, *n* (%)							
Lowering blood pressure	3842 (20.3)	19 (19.2)	99 (21.8)	1196 (17.8)	848 (17.8)	975 (25.0)	705 (23.2)
Heart	1292 (6.8)	3 (3.0)	37 (8.1)	398 (5.9)	264 (5.5)	361 (9.3)	229 (7.5)

Data are presented as median (IQR) unless otherwise specified as numbers (%).

BMI, body mass index; DANHES, Danish Health Examination Survey; MVPA, Moderate to vigorous leisure time physical activity; *N*, sample size.

a Daily smoking duration in years refers to current and former smokers [missing *n*/total *n* = 962/12 690 (females); missing *n*/total *n* = 438/10 532 (males)].

b Time since quitting daily smoking in years for former and occasional smokers only [missing *n*/total *n* = 200/9167 (females); missing *n*/total *n* = 111/7801 (males)].

Supplementary Table S3 presents the characteristics of participants excluded due to missing data. Excluded participants were older, had lower education levels, included more daily smokers and abstainers, and reported higher physical activity. Gender and BMI distribution was similar between included and excluded participants.

### Drinking pattern classes and CHD risk

Males with the frequent-heavy drinking pattern had a lower risk of CHD than those in the low drinking pattern after adjusting for potential confounders and mediators [hazard ratio (HR): 0.85, 95% CI: 0.73–0.99]. Both overall and female current abstainers showed a higher risk of CHD compared to low drinkers (Table 2). Interaction analysis between drinking patterns and sex in relation to CHD was not significant (*P*-interaction: .878). When the analysis was restricted to participants who reported stable alcohol consumption over the previous 5 years, the first 2 years of follow-up were excluded or participants who were on heart medication at baseline were not considered, the comparison in males between frequent-heavy drinking pattern and low drinking pattern were no longer significant (*P* values: .134, *P* values: .067, *P* values: .065, respectively). This inverse association was neither observed in analyses restricted to never-smokers, suggesting residual confounding by smoking or related factors rather than a true protective effect (Supplementary Table S4).

**Table 2:** Hazard ratios (95% CI) of CHD and stroke according to drinking patterns.

	Non-drinkers		Latent class groups			
	Lifetime abstainers	Current abstainers	Low	Low-to-moderate	Moderate-regular	Frequent-heavy
**CHD**						
**Females**						
Cases, *n*	21	65	164	451	308	154
Person-years	3727	10 155	38 835	136 237	84 381	34 070
HR (95% CI)						
Model 1	1.01 (0.64–1.59)	1.43 (1.07–1.91)	1 [Ref.]	0.86 (0.72–1.03)	0.82 (0.68–0.99)	0.90 (0.72–1.13)
Model 2	1.06 (0.67–1.68)	1.35 (1.01–1.80)	1 [Ref.]	0.93 (0.77–1.11)	0.87 (0.72–1.06)	0.89 (0.71–1.11)
Model 3	1.06 (0.67–1.67)	1.39 (1.04–1.85)	1 [Ref.]	0.98 (0.81–1.17)	0.96 (0.79–1.16)	0.97 (0.77–1.21)
**Males**						
Cases, *n*	10	50	533	361	387	248
Person-years	1054	4690	73 536	52 866	41 291	33 036
HR (95% CI)						
Model 1	1.19 (0.64–2.23)	1.36 (1.02–1.82)	1 [Ref.]	0.93 (0.81–1.06)	0.89 (0.78–1.02)	0.95 (0.82–1.10)
Model 2	1.18 (0.63–2.20)	1.24 (0.92–1.66)	1 [Ref.]	0.95 (0.83–1.09)	0.90 (0.79–1.03)	0.89 (0.76–1.04)
Model 3	1.25 (0.67–2.34)	1.21 (0.90–1.62)	1 [Ref.]	0.95 (0.83–1.09)	0.93 (0.81–1.06)	0.85 (0.73–0.99)
**All**						
Cases, *n*	31	115	697	812	695	402
Person-years	4781	14 844	112 370	189 103	125 672	67 106
HR (95% CI)						
Model 1	1.10 (0.77–1.58)	1.43 (1.17–1.74)	1 [Ref.]	0.91 (0.82–1.01)	0.86 (0.78–0.96)	0.94 (0.83–1.06)
Model 2	1.11 (0.77–1.59)	1.31 (1.07–1.60)	1 [Ref.]	0.94 (0.85–1.04)	0.88 (0.79–0.99)	0.90 (0.79–1.02)
Model 3	1.09 (0.76–1.57)	1.29 (1.05–1.57)	1 [Ref.]	0.95 (0.85–1.05)	0.92 (0.83–1.03)	0.89 (0.78–1.01)
**Stroke**						
Females						
Cases, *n*	21	46	130	379	262	256
Person-years	3766	10 282	39 258	137 162	85 016	34 291
HR (95% CI)						
Model 1	1.20 (0.76–1.90)	1.31 (0.94–1.83)	1 [Ref.]	0.98 (0.80–1.19)	0.93 (0.75–1.15)	1.22 (0.96–1.54)
Model 2	1.22 (0.77–1.95)	1.27 (0.91–1.78)	1 [Ref.]	1.04 (0.85–1.28)	0.99 (0.80–1.22)	1.20 (0.94––1.52)
Model 3	1.23 (0.77–1.95)	1.27 (0.91–1.78)	1 [Ref.]	1.06 (0.87–1.30)	1.01 (0.81–1.25)	1.21 (0.95–1.54)
**Males**						
Cases, *n*	5	32	332	231	292	200
Person-years	1099	4791	74 922	53 962	41 986	33 504
HR (95% CI)						
Model 1	0.88 (0.36–2.12)	1.45 (1.01–2.09)	1 [Ref.]	0.99 (0.83–1.17)	0.99 (0.84–1.16)	1.29 (1.08–1.54)
Model 2	0.88 (0.36–2.12)	1.34 (0.93–1.93)	1 [Ref.]	1.02 (0.86–1.21)	1.01 (0.86–1.19)	1.26 (1.05–1.51)
Model 3	0.87 (0.36–2.10)	1.33 (0.92–1.92)	1 [Ref.]	1.02 (0.86–1.21)	1.01 (0.86–1.19)	1.23 (1.03–1.48)
**All**						
Cases, *n*	26	78	462	610	554	356
Person-years	4865	15 073	114 180	191 123	127 001	67 795
HR (95% CI)						
Model 1	1.14 (0.77–1.70)	1.38 (1.09–1.76)	1 [Ref.]	1.00 (0.88–1.13)	0.97 (0.86–1.10)	1.26 (1.10–1.45)
Model 2	1.15 (0.77–1.71)	1.30 (1.02–1.66)	1 [Ref.]	1.03 (0.91–1.17)	1.00 (0.88–1.14)	1.23 (1.07–1.42)
Model 3	1.15 (0.77–1.71)	1.29 (1.01–1.64)	1 [Ref.]	1.04 (0.91–1.17)	1.01 (0.89–1.14)	1.20 (1.04–1.39)

HRs and 95% CI were calculated with the use of Cox proportional hazard regression model.

Model 1: age (continuous); Model 2: model 1 + smoking status (never, past, occasional, daily 1–15/day, daily ≥15/day), married or living with a partner (yes, no), education (<10 years, 10–12 years, 13–14 years, ≥15 years), occupation (working, unemployed, student, retired, other), leisure time physical activity (vigorous, moderate, light, sedentary), diet (frequent, infrequent); Model 3: model 2 + BMI (continuous and squared), diabetes (yes, no or missing), hypertension (yes now, yes previously, no or missing), lowering blood pressure medication (yes, no or missing), heart medication (yes, no or missing). The model 3 for females was additionally adjusted for menopause (peri- and postmenopausal, premenopausal, not known or missing) and for hormone replacement therapy (yes now, yes earlier, never or missing). Model for all cohort were additionally adjusted for sex (female, male). Ref., reference category.

### Drinking pattern classes and stroke risk

Males in frequent-heavy drinking pattern had a 23% higher risk of suffering a stroke (HR: 1.23; 95% CI: 1.03–1.48) compared to a low drinking pattern. In the overall study population, stroke risk was 20% higher for frequent binge drinkers and 29% higher for current abstainers, compared to low drinkers (Table 2). The association between drinking patterns and stroke risk did not differ meaningfully by sex (*P*-interaction: .983). Sensitivity analysis revealed consistent results, but when limited to participants with stable alcohol use over the past 5 years, females with a frequent binge drinking pattern had a 34% higher risk of stroke compared to low drinkers (HR: 1.34; 95% CI: 1.01–1.79) and no significant difference was observed in males (HR: 1.12; 95% CI: 0.90–1.41). The wide IC and limited number of cases among never-smokers (*n* = 780/21 195) reduced precision, so the absence of a significant association in this group should be interpreted cautiously (Table S4).

### Drinking pattern classes and all-cause mortality risk

Males who followed a low-to-moderate drinking pattern had a lower risk of all-cause mortality (HR: 0.871; 95% CI: 0.760–0.999; *P* values: .048), while current abstainers had a higher risk (HR: 1.64; 95% CI: 1.29–2.10) compared to low-level drinkers (Table 3). There was a significant interaction between males and females in how drinking pattern related to mortality (*P*-interaction: .048). In males with stable drinking habits over the past 5 years, frequent-heavy drinking pattern was linked with higher risk of death compared to the low drinkers (HR: 1.19; 95%CI: 1.00–1.42). Among females not using heart medication at baseline, moderate-regular drinkers had a lower risk of death compared to low drinkers (HR: 0.84; 95%CI: 0.71–0.99). The remaining results demonstrated consistency after conducting sensitivity analyses. In addition, similar risk patterns were seen in both the fully adjusted model and the never-smoker analysis, suggesting limited residual confounding by smoking, though uncertainty remains (Supplementary Table S4).

**Table 3:** Hazard ratios (95% CI) of all-cause mortality according to drinking patterns.

	Non-drinkers		Latent class groups			
	Lifetime abstainers	Current abstainers	Low	Low-to-moderate	Moderate-regular	Frequent-heavy
**Females**						
Cases, *n*	39	89	275	602	421	265
Person-years	3854	10 494	39 816	139 005	86 284	35 034
HR (95% CI)						
Model 1	0.87 (0.62–1.22)	1.17 (0.92–1.49)	1 [Ref.]	0.81 (0.70–0.93)	0.74 (0.64–0.86)	1.01 (0.86–1.20)
Model 2	0.95 (0.67–1.33)	1.11 (0.87–1.41)	1 [Ref.]	0.92 (0.79–1.06)	0.85 (0.73–1.00)	1.02 (0.86–1.21)
Model 3	0.94 (0.67–1.32)	1.07 (0.84–1.36)	1 [Ref.]	0.92 (0.80–1.07)	0.87 (0.74–1.02)	1.04 (0.87–1.23)
**Males**						
Cases, *n*	10	73	595	326	593	331
Person-years	1124	4964	76 608	55 085	43 385	34 482
HR (95% CI)						
Model 1	0.91 (0.49–1.70)	1.98 (1.56–2.53)	1 [Ref.]	0.84 (0.73–0.96)	1.01 (0.90–1.14)	1.29 (1.12–1.48)
Model 2	0.98 (0.52–1.82)	1.65 (1.29–2.11)	1 [Ref.]	0.87 (0.76–0.99)	1.00 (0.89–1.13)	1.13 (0.98–1.30)
Model 3	0.98 (0.53–1.84)	1.64 (1.29–2.10)	1 [Ref.]	0.87 (0.76–1.00)	1.01 (0.90–1.14)	1.12 (0.97–1.29)
**All**						
Cases, *n*	49	162	870	928	1014	596
Person-years	4978	15 458	116 424	194 091	129 670	69 516
HR (95% CI)						
Model 1	0.96 (0.72–1.28)	1.53 (1.30–1.82)	1 [Ref.]	0.87 (0.79–0.96)	0.92 (0.84–1.00)	1.18 (1.07–1.32)
Model 2	1.01 (0.75–1.35)	1.33 (1.12–1.58)	1 [Ref.]	0.93 (0.84–1.02)	0.95 (0.87–1.05)	1.08 (0.97–1.21)
Model 3	1.00 (0.75–1.34)	1.30 (1.10–1.54)	1 [Ref.]	0.93 (0.84–1.02)	0.97 (0.88–1.06)	1.09 (0.98–1.21)

HRs and 95% CI were calculated with the use of Cox proportional hazard regression model.

Model 1: age (continuous); Model 2: model 1 + smoking status (never, past, occasional, daily 1–15/day, daily ≥15/day), married or living with a partner (yes, no), education (<10 years, 10–12 years, 13–14 years, ≥15 years), occupation (working, unemployed, student, retired, other), leisure time physical activity (vigorous, moderate, light, sedentary), diet (frequent, infrequent); Model 3: model 2 + BMI (continuous and squared), diabetes (yes, no or missing), hypertension (yes now, yes previously, no or missing), lowering blood pressure medication (yes, no or missing), heart medication (yes, no, or missing). Model for all cohort were additionally adjusted for sex (female, male).

Ref., reference category.

## Discussion

We identified four distinct drinking patterns in those Danish population who reached middle-age: low, low-to-moderate, moderate-regular, and frequent-heavy. The predominant drinking pattern was the low drinking class among males and the low-to-moderate drinking class among females, both referring to a median of 3 (2–5) drinks per week. Median levels of alcohol consumption where in consonance with the values reported in other Danish cohorts [25]. Frequent-heavy drinkers had a 20% (4–39) higher risk of stroke compared with low drinkers. Similarly, males who reported a 5-year stable pattern of frequent-heavy drinking exhibited an elevated risk of mortality compared with low drinkers. In contrast, males in the low-to-moderate group and females not taking heart medication who were allocated to the moderate-regular drinking pattern might have a lower risk of all-cause mortality compared with low drinkers. The present analysis yielded inconclusive results regarding the association between drinking patterns and CHD risk. The lack of statistical significance across sensitivity analyses suggests that factors other than frequent heavy drinking may explain the initially observed association, as well as, potential residual confounding from smoking. In addition, findings highlighted the importance of distinguishing between lifetime and current abstainers. Abstainers were not an appropriate reference group due to their diverse sociodemographic and clinical characteristics, whereas low drinkers were more suitable.

The findings highlight LCA as a valuable tool in alcohol research, offering detailed insights into individual-drinking patterns. Unlike traditional methods, LCA identifies distinct behavior-based groups that reflect cultural and regional differences. In middle-aged Danes, the predominant drinking pattern consisted of a median of 3 (2–5) drinks per week [36 (24-60) g of alcohol/week], less than one or up to 2 days per week, primarily wine, consumed socially during meals and in a non-binge manner. Frequent-heavy drinking was more common in males. Females had lower alcohol intake across all classes, a result consistent with evidence that both the benefits and risks of alcohol on CHD occur at lower consumption levels in females [26]. Drinking patterns are linked to lifestyle and demographic factors, including smoking, diet, education and occupation. Previous studies found that alcohol consumption patterns differ by lifestyle factors, with higher education associated with greater intake among Danish postmenopausal women [13] and a Mediterranean drinking pattern linked to less smoking and healthier diets [18] Epstein *et al.* (1995) identified four harmful drinking patterns and illustrated distinct sociodemographic traits [27]. Beyond epidemiological evidence, a Danish randomized trial on psychosocial alcohol therapy demonstrated that alcohol-related interventions could be more effective when the messaging matched participants’ self-image [28]. These findings suggest that policy and intervention strategies may be more effective if tailored to specific drinking profiles—for instance, by addressing the social acceptability of binge drinking, regulating targeted alcohol marketing, or reducing social isolation.

The study found no increased risk among low-to-moderate or moderate-regular drinkers compared to low drinkers, suggesting a threshold above which the adverse associations emerge and supporting guidelines recommending a maximum intake of 100 grams per week for drinkers, and making hard to tease apart drinking pattern from amount [7]. In that sense, Tolstrup *et al.* (2006) reported that among women, the amount of alcohol mattered more for CHD risk, whereas in men frequency appears to play a greater role [16]. Overall, low-to-moderate alcohol consumption (1–2 drinks/day) has been linked with lower CHD [29, 30], but might be direct associated with stroke risk [29–31]. High adherence to a traditional Mediterranean drinking pattern was linked to a lower overall mortality than abstaining, especially when considering the pattern rather than moderate intake alone (45% vs. 31%) [18]. Similarly, a recent study showed a lower excess mortality associated with alcohol in those who preferred wine and drank only with meals [17]. Hisamatsu *et al.* (2022) found an inverse association between alcohol intake and CHD risk in Japanese males without coronary artery calcification burden [32], suggesting that the positive associations may involve non-atherosclerotic pathways, such as platelet aggregation [33, 34], endothelial function [35], inflammation [36], or myocyte resistance to ischemic injury [31]. While the scientific community concurs that alcohol consumption cannot be recommended for prevention, understanding how it might offer some protection remains valuable.

The study’s strengths include a large sample size, long follow-up, use of register-based data, and a detailed individualized classification of drinking patterns and sociodemographic description. Lifetime abstainers and current abstainers represented a smaller proportion of our cohort. The DANHES cohort was established in 2008, when the proportion of abstainers in Denmark was relatively low [37]. Indeed, a report from the Joint Action on Reducing Alcohol-Related Harm (RARHA) identified Denmark as the country with the lowest percentage of abstainers in the past year [38]. The present analysis also adopted a sex-specific approach and adjusted for a comprehensive collection of confounding variables.

Limitations include potential bias from self-reported alcohol use, cultural and geographical influences limiting generalizability, and reliance on a single data collection. We restricted the analysis because, after excluding individuals with missing alcohol data, pregnancy or <6 months childbirth, or baseline CHD or stroke, few participants younger than 40 years had the outcomes (CHD: *n* = 108; stroke: *n* = 104; death: *n* = 86). Therefore, our results apply only to individuals aged 40 years or older. It is also worth noting that a proportion of heavy drinkers may have been excluded from the analysis due to premature death. In addition, the study population may have been healthier than the background population, and the inclusion of only participants with complete data may have implications for selection bias and generalizability of the present results. Regarding self-reporting bias, a more accurate estimate of alcohol consumption (e.g. biomarkers of alcohol intake) could help reduce residual confounding arising from exposure misclassification. However, because exposure information was collected before the outcomes occurred, any misclassification is expected to be non-differential. In addition, the drinking patterns and other confounders, such as smoking, may change during the follow-up, introducing exposure misclassification over time and potentially changing the observed associations. Notably, the study provides strong representation of middle-aged females, a group often underrepresented in cardiovascular research despite their substantial burden of CVD [39]. Sex-specific diagnostic biases and poorer stroke outcomes in females may also be reflected in our study’s case numbers [21, 40].

In conclusion, four drinking patterns were identified in this middle-aged cohort, beyond the lifetime and current abstainer groups. Frequent-heavy drinking, often alone at home and not always within meals, was associated with a higher risk of stroke and all-cause mortality compared to low drinkers. Current abstainers were at high risk for adverse outcomes such as stroke, CHD and all-cause mortality compared with low-drinkers. Overall, the use of LCA supports the need and facilitates the implementation of targeted interventions and context-aware public health policies to reduce alcohol-related harm in the middle-aged Danish adults.

## Supplementary Material

ckag162_Supplementary_Data

## Data Availability

In accordance with the Danish Act on Processing of Personal Data and the requirement for informed consent from the participants, study data cannot be made available in a public repository and cannot be obtained upon request. Key points Among 46 141 middle-aged participants followed for a median of 11.8 years, four distinct drinking patterns were identified (labeled as “low drinkers,” “low-to-moderate drinkers,” “moderate-regular drinkers,” and “frequent-heavy drinkers”). The more frequent drinking pattern for males (36.4%) and females (46.2%) was characterized by a median of 3 (2–5) drinks per week, consumed on fewer than one or up to 2 days per week, primarily wine, socially during meals, and in a non-binge manner. Frequent-heavy drinking was associated with increased risks of stroke, whereas individuals in the low-to-moderate male drinking class and the moderate-regular female drinking class not under heart medication might have a lower risk of all-cause mortality than low-level drinkers. Alcohol policy recommendations and targeted interventions may be more effective if guided by individual sociodemographic profiles and drinking behaviors.

## References

[1] Wood AM , Kaptoge S, Butterworth A et al Risk thresholds for alcohol consumption: combined analysis of individual-participant data for 599 912 current drinkers in 83 prospective studies. Lancet 2018;391:1513–23. 10.1016/S0140-6736(18)30134-X 29676281 PMC5899998

[2] Fernández-Solà J. Cardiovascular risks and benefits of moderate and heavy alcohol consumption. Nat Rev Cardiol 2015;12:576–87. 10.1038/nrcardio.2015.91 26099843

[3] Thompson PL. J‐curve revisited: cardiovascular benefits of moderate alcohol use cannot be dismissed. Med J Aust 2013;198:419–22. 10.5694/mja12.10922 23641990

[4] Carr S , Bryazka D, McLaughlin SA et al A burden of proof study on alcohol consumption and ischemic heart disease. Nat Commun 2024;15:4082. 10.1038/s41467-024-47632-7 38744810 PMC11094064

[5] Kassaw NA , Zhou A, Mulugeta A et al Alcohol consumption and the risk of all-cause and cause-specific mortality—a linear and nonlinear Mendelian randomization study. Int J Epidemiol 2024;53:dyae046. 10.1093/ije/dyae046 PMC10951973 38508868

[6] Bryazka D , Reitsma MB, Griswold MG et al Population-level risks of alcohol consumption by amount, geography, age, sex, and year: a systematic analysis for the global burden of disease study 2020. Lancet 2022;400:185–235. 10.1016/S0140-6736(22)00847-9 35843246 PMC9289789

[7] Visseren FLJ , MacH F, Smulders YM et al 2021 ESC guidelines on cardiovascular disease prevention in clinical practice. Eur Heart J 2021;42:3227–337. 10.1093/eurheartj/ehab484 34458905

[8] Sundhedsstyrelsen. Teknisk-Notat-Om-Risikovurdering-Ved-Alkoholindtag. 2025. https://www.sst.dk/media/djfkgu4x/april-2023-teknisk-notat-om-risikovurdering-ved-alkoholindtag.pdf

[9] National Academies of Sciences, Engineering, and Medicine; Health and Medicine Division; Food and Nutrition Board; Committee, on, Review of Evidence on Alcohol and Health, Stone KB, Calonge BN. *Review of Evidence on Alcohol and Health*. Washington, DC: National Academies Press, 2025. 40393408

[10] Estruch R , Hendriks HFJ. Associations between low to moderate consumption of alcoholic beverage types and health outcomes: a systematic review. Alcohol Alcohol 2022;57:176–84. 10.1093/alcalc/agab082 34897368 PMC8919407

[11] Larsen BA , Klinedinst BS, Le ST et al Beer, wine, and spirits differentially influence body composition in older white adults—a United Kingdom biobank study. Obes Sci Pract 2022;8:641–56. 10.1002/osp4.598 36238230 PMC9535674

[12] Krittanawong C , Isath A, Rosenson RS et al Alcohol consumption and cardiovascular health. Am J Med 2022;135:1213–30.e3. 10.1016/j.amjmed.2022.04.021 35580715 PMC9529807

[13] Antoniussen CS , Proust-Lima C, Ibsen DB et al Alcohol consumption trajectories and risk of breast cancer among postmenopausal women: a Danish cohort study. Eur J Epidemiol 2024;39:1353–62. 10.1007/s10654-024-01179-5 39630402 PMC11680656

[14] Holst C , Becker U, Jørgensen ME et al Alcohol drinking patterns and risk of diabetes: a cohort study of 70,551 men and women from the general Danish population. Diabetologia 2017;60:1941–50. 10.1007/s00125-017-4359-3 28748324

[15] Sato KK , Hayashi T, Uehara S et al Drinking pattern and risk of chronic kidney disease: the Kansai healthcare study. Am J Nephrol 2014;40:516–22. 10.1159/000370051 25531762

[16] Tolstrup J , Jensen MK, Tjønneland A et al Prospective study of alcohol drinking patterns and coronary heart disease in women and men. BMJ 2006;332:1244–8. 10.1136/bmj.38831.503113.7C 16672312 PMC1471902

[17] Ortolá R , Sotos-Prieto M, García-Esquinas E et al Alcohol consumption patterns and mortality among older adults with health-related or socioeconomic risk factors. JAMA Netw Open 2024;7:e2424495. 10.1001/jamanetworkopen.2024.24495 39133491 PMC11320169

[18] Gea A , Bes-Rastrollo M, Toledo E et al Mediterranean alcohol-drinking pattern and mortality in the SUN (Seguimiento Universidad de Navarra) project: a prospective cohort study. Br J Nutr 2014;111:1871–80. 10.1017/S0007114513004376 24480368

[19] Mostofsky E , Chahal HS, Mukamal KJ et al Alcohol and immediate risk of cardiovascular events. Circulation 2016;133:979–87. 10.1161/CIRCULATIONAHA.115.019743 26936862 PMC4783255

[20] Lanza ST , Cooper BR. Latent class analysis for developmental research. Child Dev Perspect 2016;10:59–64. 10.1111/cdep.12163 31844424 PMC6914261

[21] Vogel B , Acevedo M, Appelman Y et al The lancet women and cardiovascular disease commission: reducing the global burden by 2030. Lancet 2021;397:2385–438. 10.1016/S0140-6736(21)00684-X 34010613

[22] Plant ML. The role of alcohol in women’s lives: a review of issues and responses. J Subst Use 2008;13:155–91. 10.1080/14659890802040880

[23] Eriksen L , Grønbæk M, Helge JW et al The Danish health examination survey 2007-2008 (DANHES 2007-2008). Scand J Public Health 2011;39:203–11. 10.1177/1403494810393557 21257645

[24] Saltin B , Grimby G. Physiological analysis of middle-aged and old former athletes. Circulation 1968;38:1104–15. 10.1161/01.CIR.38.6.1104 5721960

[25] Rohde JF , Ängquist L, Larsen SC et al Alcohol consumption and its interaction with adiposity-associated genetic variants in relation to subsequent changes in waist circumference and body weight. Nutr J 2017;16:51. 10.1186/s12937-017-0274-1 28841830 PMC5574083

[26] Corrao G , Rubbiati L, Bagnardi V et al Alcohol and coronary heart disease: a meta-analysis. Addiction 2000;95:1505–23. 10.1046/j.1360-0443.2000.951015056.x 11070527

[27] Epstein EE , Kahler CW, Mccrady BS et al An empirical classification of drinking patterns among alcoholics: binge, episodic, sporadic, and steady. Addict Behav 1995;20:23–41. 7785480 10.1016/0306-4603(94)00043-x

[28] Egan KK , Tjørnhøj-Thomsen T, Becker U et al Exploring reasons behind the initiation of and compliance with proactive alcohol e-therapy: a qualitative study. Drug Alcohol Depend 2025;269:112585. 10.1016/j.drugalcdep.2025.112585 39938335

[29] Ronksley PE , Brien SE, Turner BJ et al Association of alcohol consumption with selected cardiovascular disease outcomes: a systematic review and meta-analysis. BMJ 2011;342:d671. 10.1136/bmj.d671 21343207 PMC3043109

[30] Ricci C , Wood A, Muller D et al Alcohol intake in relation to non-fatal and fatal coronary heart disease and stroke: EPIC-CVD case-cohort study. BMJ 2018;361:k934. 10.1136/bmj.k934 29844013 PMC5972779

[31] Collins MA , Neafsey EJ, Mukamal KJ et al Alcohol in moderation, cardioprotection, and neuroprotection: epidemiological considerations and mechanistic studies. Alcohol Clin Exp Res 2009;33:206–19. 10.1111/j.1530-0277.2008.00828.x 19032583 PMC2908373

[32] Hisamatsu T , Miura K, Tabara Y et al Alcohol consumption and subclinical and clinical coronary heart disease: a Mendelian randomization analysis. Eur J Prev Cardiol 2022;29:2006–14. 10.1093/eurjpc/zwac156 35907253

[33] Rimm EB , Williams P, Fosher K et al Moderate alcohol intake and lower risk of coronary heart disease: meta-analysis of effects on lipids and haemostatic factors. BMJ 1999;319:1523–8. 10.1136/bmj.319.7224.1523 10591709 PMC28294

[34] Mukamal KJ , Jensen MK, Grønbaek M et al Drinking frequency, mediating biomarkers, and risk of myocardial infarction in women and men. Circulation 2005;112:1406–13. 10.1161/CIRCULATIONAHA.105.537704 16129796

[35] Sacanella E , Badía E, Nicolás JM et al Differential effects of moderate or heavy alcohol consumption on circulating adhesion molecule levels. Thromb Haemost 2002;88:52–5. 12152678

[36] Imhof A , Froehlich M, Brenner H et al Effect of alcohol consumption on systemic markers of inflammation. Lancet 2001;357:763–7. 10.1016/S0140-6736(00)04170-2 11253971

[37] Bloomfield K , Grittner U, Rasmussen HB et al Socio-demographic correlates of alcohol consumption in the Danish general population. Scand J Public Health 2008;36:580–8. 10.1177/1403494808089648 18775814

[38] Bedyńska S , Brzózka K, Moskalewicz J et al Comparative monitoring of alcohol epidemiology across the EU. Baseline assessment and suggestions for future action. Synthesis Report. PARPA – The State Agency for Prevention of Alcohol Related Problems, Warsaw. 2016.

[39] Global Burden of Disease Collaborative Network. Global Burden of Disease Study 2019 (GBD 2019) Results. Seattle, WA: Institute for Health Metrics and Evaluation, 2020. https://gbd2019.healthdata.org/gbd-compare/ (27 January 2025, date last accessed).

[40] Wenger NK. The feminine face of heart disease 2024. Circulation 2024;149:489–91. 10.1161/CIRCULATIONAHA.123.064460 38346107

