# Supplementary material for: Identifying drinking patterns in a middle-aged population and their association with coronary heart disease, stroke, and all-cause mortality risk: the Danish Health Examination Survey
Source: Eur J Public Health. 2026 Sep 2;36(5):ckag162. doi: 10.1093/eurpub/ckag162 (PMC13537461; doi:10.1093/eurpub/ckag162)
Supplement: Supplementary file 1 [file ckag162_supplementary_data.zip › Supplementary_Data/ejph-2025-08-om-0728-File003.docx]

**Supplemental Online Tables**

**Supplementary Table S1.** Fit statistics for latent class models

**Supplementary Table S2.** Drinking pattern characteristics across drinking pattern latent classes

**Supplementary Table S3.** Sociodemographic characteristics of included and excluded participants due to missing covariate data

**Supplementary Table S4.** Comparison of hazard ratios for CHD, stroke and all-cause mortality from the fully adjusted model and stratified analyses by smoking status (never and current smokers)

Supplementary Table S1. Fit statistics for latent class models

| **Models** | | **LL** | **AIC** | **BIC** | **Smallest Class Count (n)** | **Smallest Class Size (%)** | **Entropy** | **ALCPP** |
| --- | --- | --- | --- | --- | --- | --- | --- | --- |
| **Females** | |  |  |  |  |  |  |  |
|  | 2 Class | -104,012 | 8,630 | 8,818 | 10,176 | 39.3 | 0.883 | 0.972 |
|  | 3 Class | -102,434 | 5,498 | 5,783 | 7,674 | 29.6 | 0.811 | 0.922 |
|  | **4 Class** | **-101,408** | **3,470** | **3,854** | **3,057** | **11.8** | **0.901** | **0.956** |
|  | 5 Class | -100,683 | 2,044 | 2,526 | 1,541 | 6.0 | 0.839 | 0.909 |
|  | 6 Class | -100,419 | 1,540 | 2,119 | 1,451 | 5.6 | 0.810 | 0.871 |
| **Males** | |  |  |  |  |  |  |  |
|  | 2 Class | -90,013 | 8,520 | 8,700 | 7,885 | 42.8 | 0.826 | 0.947 |
|  | 3 Class | -88,303 | 5,125 | 5,399 | 4,081 | 22.2 | 0.847 | 0.929 |
|  | **4 Class** | **-87,354** | **3,250** | **3,618** | **3,045** | **16.5** | **0.825** | **0.912** |
|  | 5 Class | -86,951 | 2,470 | 2,932 | 2,761 | 15.0 | 0.791 | 0.865 |
|  | 6 Class | -86,714 | 2,019 | 2,574 | 1,935 | 10.5 | 0.760 | 0.836 |

Note. LL = Log-Likelihood; AIC = Akaike Information Criterion; BIC = Bayesian Information Criterion; ALCPP = Average latent class posterior probability.

**Supplementary Table S2.** Drinking pattern characteristics across drinking pattern latent classes

|  | **Low** | **Low-to-moderate** | **Moderate regular** | **Frequent -heavy** |
| --- | --- | --- | --- | --- |
| **Females*, n*** | 3,456 | 11,952 | 7,434 | 3,057 |
| Alcohol amount*, drinks/week* | 0 (0-1) | 3 (2-5) | 9 (7-11) | 17 (15-21) |
| Frequency of alcohol drinking*, n (%)* |  |  |  |  |
| <1 day/week | 2,942 (86) | 4,708 (39) | 329 (4) | 53 (2) |
| 1-2 day/week | 398 (12) | 5,696 (48) | 1,640 (22) | 100 (3) |
| 3-4 day/week | 92 (3) | 1,527 (13) | 2,859 (39) | 434 (14) |
| 5-7 day/week | 0 (0) | 0 (0) | 2,593 (35) | 2,459 (81) |
| Binge episodes >1/month*, n (%)* | 60 (1.7) | 407 (3.4) | 1,181 (15.9) | 1,298 (42.5) |
| Proportion of total alcohol intake*, %* |  |  |  |  |
| Beer | 0 (0-0) | 0 (0-25) | 8 (0-23) | 0 (0-17) |
| Wine | 0 (0-0 | 100 (67-100) | 86 (67-100) | 90 (67-100) |
| Spirits | 0 (0-33) | 0 (0-0 | 0 (0-10) | 0 (0-8) |
| Drinking within meals*, n (%)* |  |  |  |  |
| None | 608 (17.8) | 437 (3.7) | 234 (3.2) | 129 (4.2) |
| ¼ or ½ | 506 (14.8) | 1,410 (11,8) | 1,135 (15,3) | 827 (27.1) |
| ¾ | 528 (15.5) | 2,605 (21.8) | 2,363 (31.8) | 985 (32.3) |
| All | 1,769 (51.9) | 7,480 (62.7) | 3,688 (49.7) | 1,107 (36.3) |
| Drinking often alone*, n (%)* | 160 (4.7) | 785 (6.6) | 1,138 (15.3) | 868 (28.4) |
| **Males*, n*** | 6,701 | 4,776 | 3,896 | 3,045 |
| Alcohol amount*, drinks/week* | 3 (2-5) | 10 (8-12) | 14 (10-18) | 24 (19-32) |
| Frequency of alcohol drinking*, n (%)* |  |  |  |  |
| <1 day/week | 2,953 (44) | 107 (2) | 19 (0) | 61 (2) |
| 1-2 day/week | 2,919 (44) | 1,815 (38) | 0 (0) | 126 (4) |
| 3-4 day/week | 820 (12) | 2,842 (60) | 8 (0) | 465 (15) |
| 5-7 day/week | 0 (0) | 0 (0) | 3,849 (99) | 2,391 (79) |
| Binge episodes >1/month*, n (%)* | 377 (5.6) | 1,832 (38.4) | 306 (7.9) | 2,947 (96.8) |
| Proportion of total alcohol intake*, (%)* |  |  |  |  |
| Beer | 33 (0-60) | 27 (13-44) | 22 (0-50) | 42 (14-69) |
| Wine | 50 (0-75) | 60 (43-80) | 62 (33-88) | 46 (21-74) |
| Spirits | 0 (0-0) | 0 (0-14) | 0 (0-14) | 0 (0-13) |
| Drinking within meals*, n (%)* |  |  |  |  |
| None | 711 (10.6) | 148 (3.1) | 171 (4.4) | 257 (8.4) |
| ¼ or ½ | 1,567 (23,4) | 1,336 (28.0) | 763 (19.6) | 1,572 (51,7) |
| ¾ | 1,956 (29.3) | 2,175 (45.6) | 1,211 (31.2) | 1,011 (33.2) |
| All | 2,453 (36.7) | 1,109 (23.3) | 1,741 (44.8) | 202 (6.6) |
| Drinking often alone*, n (%)* | 525 (7.9) | 480 (10.1) | 845 (21.7) | 1,297 (42.6) |

Data are presented as median (IQR) and n (%)

**Supplementary Table S3.** Sociodemographic characteristics of included and excluded participants due to missing covariate data

| Covariate | Included (n=46,143) | Excluded (n=4,434) |
| --- | --- | --- |
| Age*, years* | 56.4 (56.3-56.5) | 60.1 (59.7-60.3) |
| Females, *n* (%) | 27,172 (58.9) | 2,650 (59.8) |
| Education >14 years, *n* (%) | 19,493 (42.2) | 529 (31.4) |
| Smoking status, *n* (%) |  |  |
| Current smokers | 7,882 (17.1) | 908 (20.8) |
| Former smokers | 17,066 (37.0) | 1,611 (36.8) |
| Never smokers | 21,195 (45.9) | 1,857 (42.4) |
| Drinking status, *n* (%) |  |  |
| Lifetime abstainer | 443 (1.0) | 108 (2.4) |
| Current abstainer | 1,381 (3.0) | 227 (5.1) |
| Drinker | 44,319 (96.1) | 4,099 (92.4) |
| Physical activity, *n* (%) |  |  |
| Sendentary | 957 (2.1) | 65 (2.2) |
| Light | 10.6 (23.1) | 550 (18.3) |
| Moderate | 27,988 (60.7) | 1,837 (61.0) |
| Vigorous | 6,551 (14.2) | 561 (18.6) |
| BMI, *kg/m^2^* | 25.3 (25.3-25.4) | 25.4 (25.3-25.6) |

Data are presented as median (IQR) unless otherwise specified as numbers (%). BMI, body mass index. Missing data in excluded: education (n=2,748); smoking status (n=58); physical activity (n=1,421); BMI (n=1,620).

**Supplementary Table S4.** Comparison of hazard ratios for CHD, stroke and all-cause mortality from the fully adjusted model and stratified analyses by smoking status (never and current smokers)

| **Drinking pattern** | | **Model 3**  **(fully adjusted)^1^** | **Never smokers^2^** | **Current smokers^2^** |  |
| --- | --- | --- | --- | --- | --- |
| **CHD** | | | | |  |
| **Females (cases/total N)** | | 1,163/27,170 | 481/13,414 | 203/4,387 |  |
| Low-drinkers | | 1.00 (ref) | 1.00 (ref) | 1.00 (ref) |  |
| Low-moderate | | 0.98 (0.81 - 1.17) | 1.00 (0.75- 1.32) | 1.02 (0.67 - 1.56) |  |
| Moderate-regular | | 0.96 (0.79 - 1.16) | 0.95 (0.70 - 1.30) | 1.04 (0.66 - 1.65) |  |
| Frequent-heavy | | 0.97 (0.77 - 1.21) | 1.00 (0.68 - 1.49) | 1.12 (0.69 - 1.82) |  |
| Lifetime abstainers | | 1.06 (0.67 - 1.67) | 1.04 (0.57 - 1.89) | 0.89 (0.21 - 3.78) |  |
| Current abstainers | | 1.39 (1.04 - 1.85) | 1.72 (1.11 - 2.65) | 1.71 (0.94 - 3.12) |  |
| **Males (cases/total N)** | | 1,589/18,971 | 472/7,781 | 342/3,495 |  |
| Low-drinkers | | 1.00 (ref) | 1.00 (ref) | 1.00 (ref) |  |
| Low-moderate | | 0.95 (0.83 - 1.09) | 1.05 (0.83 - 1.32) | 0.93 (0.69 - 1.25) |  |
| Moderate-regular | | 0.93 (0.81 - 1.06) | 0.95 (0.74 - 1.23) | 0.90 (0.66 - 1.21) |  |
| Frequent-heavy | | 0.85 (0.73 - 0.99) | 1.01 (0.74 - 1.38) | 0.64 (0.47 - 0.87) |  |
| Lifetime abstainers | | 1.25 (0.67 - 2.34) | 1.48 (0.65 - 3.37) | 0.51 (0.07 - 3.68) |  |
| Current abstainers | | 1.21 (0.90 - 1.62) | 1.44 (0.83 - 2.49) | 1.12 (0.64 - 1.96) |  |
| **All (cases/total N)** | | 2,752/46,141 | 953/21,195 | 545/7,882 |  |
| Low-drinkers | | 1.00 (ref) | 1.00 (ref) | 1.00 (ref) |  |
| Low-moderate | | 0.95 (0.85 - 1.05) | 1.02 (0.86 - 1.21) | 0.94 (0.74 - 1.19) |  |
| Moderate-regular | | 0.92 (0.83 - 1.03) | 0.95 (0.79 - 1.16) | 0.91 (0.72 - 1.17) |  |
| Frequent-heavy | | 0.89 (0.78 - 1.01) | 1.02 (0.80 - 1.29) | 0.75 (0.58 - 0.97) |  |
| Lifetime abstainers | | 1.09 (0.76 - 1.57) | 1.15 (0.72 - 1.85) | 0.70 (0.22 - 2.20) |  |
| Current abstainers | | 1.29 (1.05 - 1.57) | 1.64 (1.18 - 2.27) | 1.33 (0.90 - 1.97) |  |
| **Stroke** | | | | |  |
| **Females (cases/total N)** | | 994/27,170 | 446/13,414 | 177/4,387 |  |
| Low-drinkers | | 1.00 (ref) | 1.00 (ref) | 1.00 (ref) |  |
| Low-moderate | | 1.01 (0.81 - 1.25) | 0.98 (0.73 - 1.31) | 1.14 (0.72 - 1.80) |  |
| Moderate-regular | | 1.06 (0.87 - 1.30) | 1.03 (0.75 - 1.41) | 0.81 (0.48 - 1.35) |  |
| Frequent-heavy | | 1.21 (0.95 - 1.54) | 0.98 (0.65 - 1.47) | 1.38 (0.84 - 2.31) |  |
| Lifetime abstainers | | 1.23 (0.77 - 1.95) | 1.60 (0.94 - 2.72) | 0.85 (0.20 - 3.68) |  |
| Current abstainers | | 1.27 (0.91 - 1.78) | 1.09 (0.64 - 1.88) | 1.66 (0.83 - 3.31) |  |
| **Males (cases/total N)** | | 1,092/18,971 | 334/7,781 | 240/3,495 |  |
| Low-drinkers | | 1.00 (ref) | 1.00 (ref) | 1.00 (ref) |  |
| Low-moderate | | 1.02 (0.86 - 1.21) | 0.80 (0.59 - 1.07) | 0.90 (0.58 - 1.39) |  |
| Moderate-regular | | 1.01 (0.86 - 1.19) | 0.98 (0.74 - 1.30) | 1.19 (0.82 - 1.74) |  |
| Frequent-heavy | | 1.23 (1.03 - 1.48) | 0.94 (0.64 - 1.39) | 1.62 (1.14 - 2.30) |  |
| Lifetime abstainers | | 0.87 (0.36 - 2.10) | 0.37 (0.56 - 3.38) | - |  |
| Current abstainers | | 1.33 (0.92 - 1.92) | 1.91 (1.07 - 3.39) | 1.97 (1.04 - 3.71) |  |
| **All (cases/total N)** | | 2,086/46,141 | 780/21,195 | 417/7,882 |  |
| Low-drinkers | | 1.00 (ref) | 1.00 (ref) | 1.00 (ref) |  |
| Low-moderate | | 1.04 (0.91 - 1.17) | 0.90 (0.74 - 1.09) | 1.09 (0.81 - 1.47) |  |
| Moderate-regular | | 1.01 (0.89 - 1.14) | 1.00 (0.81 - 1.23) | 1.09 (0.80 - 1.47) |  |
| Frequent-heavy | | 1.20 (1.04 – 1.39) | 0.95 (0.72 - 1.25) | 1.56 (1.17 - 2.07) |  |
| Lifetime abstainers | | 1.15 (0.77 - 1.71) | 1.50 (0.97 - 2.34) | 0.66 (0.16 - 2.73) |  |
| Current abstainers | | 1.29 (1.01 - 1.64) | 1.30 (0.88 - 1.92) | 1.82 (1.14 - 2.89) |  |
| **All-cause mortality** | | | | |  |
| **Females (cases/total N)** | | 1,691/27,170 | 647/13,414 | 385/4,387 |  |
| Low-drinkers | | 1.00 (ref) | 1.00 (ref) | 1.00 (ref) |  |
| Low-moderate | | 0.92 (0.80 - 1.07) | 0.88 (0.70 - 1.10) | 0.93 (0.68 - 1.27) |  |
| **Supplementary Table S4 (continued)** | | |  |  |  |
| **Drinking pattern** | **Model 3**  **(fully adjusted)^1^** | | **Never smokers^2^** | **Current smokers^2^** |  |
|  | |  |  |  |  |
| Moderate-regular | | 0.87 (0.74 - 1.02) | 0.78 (0.60 - 1.00) | 1.00 (0.72 - 1.39) |  |
| Frequent-heavy | | 1.04 (0.87 - 1.23) | 0.90 (0.65 - 1.25) | 1.24 (0.89 - 1.74) |  |
| Lifetime abstainers | | 0.94 (0.67 - 1.32) | 0.86 (0.55 - 1.33) | 0.75 (0.30 - 1.90) |  |
| Current abstainers | | 1.07 (0.84 - 1.36) | 0.74 (0.48 - 1.15) | 1.35 (0.85 - 2.15) |  |
|  | |  |  |  |  |
| **Males (cases/total N)** | | 1,928/18,971 | 449/7,781 | 482/3,495 |  |
| Low-drinkers | | 1.00 (ref) | 1.00 (ref) | 1.00 (ref) |  |
| Low-moderate | | 0.87 (0.76 - 1.00) | 0.89 (0.69 - 1.15) | 0.79 (0.58 - 1.07) |  |
| Moderate-regular | | 1.01 (0.90 - 1.14) | 0.93 (0.72 - 1.19) | 1.13 (0.88 - 1.46) |  |
| Frequent-heavy | | 1.12 (0.97 - 1.29) | 1.23 (0.90 - 1.67) | 1.32 (1.02 - 1.69) |  |
| Lifetime abstainers | | 0.98 (0.53 - 1.84) | 1.17 (0.51 - 2.68) | 0.85 (0.21 - 3.47) |  |
| Current abstainers | | 1.64 (1.29 - 2.10) | 1.01 (0.53 - 1.92) | 2.05 (1.36- 3.08) |  |
| **All (cases/total N)** | | 3,619/46,141 | 1,096/21,195 | 867/7,882 |  |
| Low-drinkers | | 1.00 (ref) | 1.00 (ref) | 1.00 (ref) |  |
| Low-moderate | | 0.93 (0.84 - 1.02) | 0.93 (0.79 - 1.10) | 0.89 (0.72 - 1.10) |  |
| Moderate-regular | | 0.97 (0.88 - 1.06) | 0.88 (0.74 - 1.05) | 1.08 (0.86 - 1.31) |  |
| Frequent-heavy | | 1.09 (0.98 - 1.21) | 1.08 (0.87 - 1.35) | 1.30 (1.07 - 1.59) |  |
| Lifetime abstainers | | 1.00 (0.75 - 1.34) | 0.99 (0.68 - 1.44) | 0.72 (0.34 - 1.55) |  |
| Current abstainers | | 1.30 (1.10 - 1.54) | 0.86 (0.60 - 1.23) | 1.69 (1.24 - 2.28) |  |

^1^Model 3: age (continuous), smoking status (never, past, occasional, daily 1-15/day, daily ≥15/day), married or living with a partner (yes, no), education (<10 years, 10-12 years, 13-14 years, ≥15 years), occupation (working, unemployed, student, retired, other), leisure time physical activity (vigorous, moderate, light, sedentary), diet (frequent, infrequent), BMI (continuous and squared), diabetes (yes, no or missing), hypertension (yes now, yes previously, no or missing), lowering blood pressure medication (yes, no or missing), heart medication (yes, no or missing). The model 3 for females was additionally adjusted for menopause (peri and postmenopausal, premenopausal, not known or missing) and for hormone replacement therapy (yes now, yes earlier, never or missing). Model for all cohort were additionally adjusted for sex (female, male).

^2^Adjusted for Model 3 except for smoking.

CHD: coronary heart disease. Ref: reference
